# Assessment of genetic diversity, population structure and relationships in Indian and non-Indian genotypes of finger millet (*Eleusine coracana* (L.) Gaertn) using genomic SSR markers

**DOI:** 10.1186/s40064-015-1626-y

**Published:** 2016-02-11

**Authors:** M. Ramakrishnan, S. Antony Ceasar, V. Duraipandiyan, N. A. Al-Dhabi, S. Ignacimuthu

**Affiliations:** Division of Plant Biotechnology, Entomology Research Institute, Loyola College, Chennai, 600 034 India; Faculty of Biological Sciences, Centre for Plant Sciences and School of Molecular and Cellular Biology, University of Leeds, Leeds, LS2 9JT UK; Department of Botany and Microbiology, Addiriyah Chair for Environmental Studies, College of Science, King Saud University, P.O.Box. 2455, Riyadh, 11451 Kingdom of Saudi Arabia; Visiting Professor Program, Deanship of Scientific Research, College of Science, King Saud University, P.O.Box. 2455, Riyadh, 11451 Kingdom of Saudi Arabia

**Keywords:** Finger millet, Genetic diversity, AMOVA, PCA, Population structure, Baysian statistics

## Abstract

We evaluated the genetic variation and population structure in Indian and non-Indian genotypes of finger millet using 87 genomic SSR primers. The 128 finger millet genotypes were collected and genomic DNA was isolated. Eighty-seven genomic SSR primers with 60–70 % GC contents were used for PCR analysis of 128 finger millet genotypes. The PCR products were separated and visualized on a 6 % polyacrylamide gel followed by silver staining. The data were used to estimate major allele frequency using Power Marker v3.0. Dendrograms were constructed based on the Jaccard’s similarity coefficient. Statistical fitness and population structure analyses were performed to find the genetic diversity. The mean major allele frequency was 0.92; the means of polymorphic alleles were 2.13 per primer and 1.45 per genotype; the average polymorphism was 59.94 % per primer and average PIC value was 0.44 per primer. Indian genotypes produced an additional 0.21 allele than non-Indian genotypes. Gene diversity was in the range from 0.02 to 0.35. The average heterozygosity was 0.11, close to 100 % homozygosity. The highest inbreeding coefficient was observed with SSR marker UGEP67. The Jaccard’s similarity coefficient value ranged from 0.011 to 0.836. The highest similarity value was 0.836 between genotypes DPI009-04 and GPU-45. Indian genotypes were placed in *Eleusine coracana* major cluster (*Ec*MC) 1 along with 6 non-Indian genotypes. AMOVA showed that molecular variance in genotypes from various geographical regions was 4 %; among populations it was 3 % and within populations it was 93 %. PCA scatter plot analysis showed that GPU-28, GPU-45 and DPI009-04 were closely dispersed in first component axis. In structural analysis, the genotypes were divided into three subpopulations (SP1, SP2 and SP3). All the three subpopulations had an admixture of alleles and no pure line was observed. These analyses confirmed that all the genotypes were genetically diverse and had been grouped based on their geographic regions.

## Background

Micronutrient deficiency has been recognized as a serious human health problem worldwide (Kanatti et al. 2014). Finger millet (*Eleusine coracana* L. Gaertn.) is a highly self-pollinating crop majorly cultivated in less developed countries of Asia and Africa. It is a good source of micronutrients like, iron and zinc. Biofortification of staple crops is a sustainable and cost-effective approach for availability of micronutrients. Biofortified cultivars of finger millet for improved micronutrients are acceptable to consumers as their adoption does not call for change in dietary habits. Analysis of genetic diversity leading to molecular breeding is a major approach for development of biofortified cultivars of finger millet.

Study of genetic diversity and population structure between genotypes has long been a major goal for crop development (Qin et al. 2009; Yang et al. 2013; Egbadzor et al. 2014; Sharma et al. 2014). India is the largest producer of finger millet and more than 34,160 cultivable genotypes are available world-wide and India alone has 22,583 genotypes; these include 9522 genotypes with National Bureau of Plant Genetic Resources (NBPGR), New Delhi, 6804 genotypes with International Crops Research Institute for the Semi-Arid Tropics (ICRISAT), Patancheru, Hyderabad and 6257 genotypes with All India Coordinated Minor Millet Project (AICMMP), Bangalore (Goron and Raizada 2015). However, only few authors have reported the genetic diversity analysis of finger millet genotypes using simple sequence repeats (SSR) markers. The numbers of genotypes used by various groups for genetic diversity analysis of finger millet genotypes using SSR markers include 79 by Dida et al. (2008), 83 by Panwar et al. (2010b), 52 by Panwar et al. (2010a) and Kumar et al. (2012), 67 by Arya et al. (2013), 103 by Nirgude et al. (2014) and 190 by Babu et al. (2014b).

SSR are tandem repeats of 2-6 base pairs which are highly polymorphic and variable in the number of repeats at a specific locus. They are widely distributed throughout the genomes in both coding and non-coding regions; they are codominant, multi-allelic, chromosome specific and highly informative genetic markers (Cho et al. 2000; Scott et al. 2000). They are amenable to high throughput genotyping, thus suitable for diversity analysis (Hua et al. 2015; Wang et al. 2011). The SSR markers have been used successfully for the evaluation of genetic diversity among several species, including finger millet (Babu et al. 2014b), foxtail millet (Kim et al. 2012; Zhao et al. 2012) and proso millet (Cho et al. 2010).

The present study aimed to assess the extent of genetic variation and population structure at the molecular level in 64 Indian, 61 non-Indian and 3 unknown origin finger millet genotypes with a view to provide data for breeding programes. In the present study, 87 genomic SSR primers were used to study the genetic variation and population structure among 128 genotypes of finger millet. Data generated through this study will be useful for breeding programes and as a resource for gene banks in future to improve the finger millet genotypes.

## Methods

### Plant materials and isolation of genomic DNA

The details of 128 finger millet genotypes and their origins are listed in Table 1. Seeds of these genotypes were obtained from the ICRISAT, Patancheru, India, University of Agricultural Sciences, Bangalore, India and Tamil Nadu Agricultural University, Coimbatore, India. The genomic DNA was isolated from all 128 genotypes (3 plants for each genotype) based on the protocol described in our previous report (Ramakrishnan et al. 2015). The purity and concentration of DNA were quantified using a Nanodrop-spectrophotometer (ND-2000,ThermoScientific, Wilmington, DE, USA) and the DNA was diluted to 50 ng/µl concentration with 0.1× TE buffer for SSR genotyping.

**Table 1:** Details of finger millet genotypes collected from different geographical regions used for the analysis of genetic diversity using genomic SSR markers

Varieties	Source country	Varieties	Source country	Varieties	Source country	Varieties	Source country
APSKK-1	India	SVK-1	India	IE-2957	Germany	IE-4795	Zimbabwe
CO- (RA) 14	India	T-CHIN-1	India	IE-3045	India	IE-4797	Maldives
CO-(NO)-1	India	T-CUM-1	India	IE-3077	India	IE-4816	India
CO-11	India	THRV-P	India	IE-3104	India	IE-5066	Senegal
CO-12	India	THRV-PP	India	IE-3317	Zimbabwe	IE-5091	Zimbabwe
CO-7	India	TRY-1	India	IE-3391	Zimbabwe	IE-5106	Zimbabwe
CO-9	India	VIJAYAWADA	India	IE-3392	Zimbabwe	IE-5201	India
GPU-26	India	VL-149	India	IE-3470	India	IE-5306	Zimbabwe
GPU-28	India	VR-708	India	IE-3475	India	IE-5367	Kenya
GPU-45	India	THRP-1	India	IE-3614	NA	IE-5537	Nepal
GPU-46	India	IE-501	India	IE-3618	NA	IE-5817	Nepal
GPU-48	India	IE-518	India	IE-3721	Uganda	IE-5870	Nepal
GPU-66	India	IE-1055	NA	IE-3945	Uganda	IE-6059	Nepal
GPU-67	India	IE-2034	India	IE-3952	Uganda	IE-6082	Nepal
HOSUR-1	India	IE-2042	India	IE-3973	Uganda	IE-6154	Nepal
HR-374	India	IE-2043	India	IE-4028	Uganda	IE-6165	Nepal
HR-911	India	IE-2217	India	IE-4057	Uganda	IE-6221	Nepal
INDOF-5	India	IE-2296	India	IE-4073	Uganda	IE-6240	Zimbabwe
INDOF-7	India	IE-2312	India	IE-4121	Uganda	IE-6294	Zimbabwe
INDOF-8	India	IE-2430	Kenya	IE-4329	Zimbabwe	IE-6326	Zimbabwe
INDOF-9	India	IE-2437	Kenya	IE-4491	Zimbabwe	IE-6337	Zimbabwe
KM-252	India	IE-2457	Kenya	IE-4497	Zimbabwe	IE-6350	Zimbabwe
KMR-301	India	IE-2572	Kenya	IE-4545	Zimbabwe	IE-6421	Uganda
L-5	India	IE-2589	USA	IE-4565	Zimbabwe	IE-6473	Uganda
M6-6	India	IE-2606	Malawi	IE-4570	Zimbabwe	IE-6514	Zimbabwe
ML-365	India	IE-2619	Malawi	IE-4622	Zimbabwe	IE-6537	Nigeria
MR-1	India	IE-2710	Malawi	IE-4646	Zimbabwe	IE-7018	Kenya
MR-2	India	IE-2790	Malawi	IE-4671	India	IE-7079	Kenya
PAIYUR-2	India	IE-2821	Nepal	IE-4673	India	IE-7320	Kenya
PES-110	India	IE-2871	Zambia	IE-4709	Burundi	KRI007-01	India
PR-202	India	IE-2872	Zambia	IE-4734	India	DPI009-04	India
RAU-8	India	IE-2911	Zambia	IE-4757	India	KRI13-11	India

### PCR amplification and silver staining

Eighty-seven genomic SSR primers with 60–70 % GC contents were used to study the genetic diversity. The PCR reactions were performed in 25 µl reaction mixture containing 50 ng each of genomic DNA, 2.5 mM MgCl_2_, 0.25 mM dNTPs, 400 nM each of forward and reverse primers and 1 U *Taq* DNA Polymerase (Genet Bio, Daejeon, Korea). The amplification was carried out in a DNA thermal cycler (Eppendorf, Gradient Thermal Cycler, Germany). The PCR was performed with an initial denaturation at 95 °C for 5 min followed by 35 cycles of 30 s denaturation at 95 °C, 30 s annealing at different temperatures based on the primer pair and 1 min extension at 72 °C with a final extension at 72 °C for 10 min. The PCR products were separated and visualized on a 6 % polyacrylamide gel followed by silver staining. The fragment sizes of the PCR products were estimated by comparison with 100 base pair (bp) and 50 bp DNA ladders; alleles size were visually scored in bp for each genotype; amplification was repeated with each primer to confirm the reproducibility of the results.

### Genetic statistics

The data were used to estimate major allele frequency, allele number, gene diversity, heterozygosity, polymorphic information content (PIC) and inbreeding coefficient using Power Marker v3.0 (Liu and Muse 2005). Dendrograms were constructed with 1000 bootstrapping values using unweighted pair-group method with arithmetic average (UPGMA) based on the Jaccard’s similarity coefficient (Jaccard 1908) using FREE TREE and TREE VIEW softwares. Analysis of the molecular variation (AMOVA) was performed using GenALEx software version 6.5 (Peakall and Smouse 2012) to test the total molecular variance among the various geographical regions, among the populations and within the populations.

### Statistical fitness analysis

To validate the cluster analysis and genetic structure, the cophenetic correlation coefficient (CCC) value was calculated using UPGMA. The distribution of populations was analyzed using Principal component analysis (PCA) which was carried out using PAST version 2 software (Hammer et al. 2001). The number of significant components to interpret from PCA was determined by both Jolliffe cut-off value and broken stick model (Jolliffe 2002).

### Population structure analysis

Analysis of the population structure and gene flow between 128 finger millet genotypes was carried out with 87 genomic SSR primers using a model-based Bayesian statistics implemented to subdivide genotypes into genetic subpopulations (SPs) using the software STRUCTURE v.2.3.4 (Pritchard et al. 2000; Ramasamy et al. 2014). In the present study, no prior knowledge was used to define SP and it was expected that number of SPs existed in the sample analyzed. Each genotype can be a member of a different subgroup (admixture model; ALPHAPROPSD = 0.20). The number of subgroups (*K*) in the population was determined by running the programme with *K* values varying from 1 to 10, with five independent runs for each *K* value. To determine most appropriate *K* value, burn-in Markov Chain Monte Carlo (MCMC) (Bayesian statistics) (Karandikar 2006) replication was set to 100,000 and data were collected over 500,000 MCMC replications in each run. The *K* value was detected using (Structure Harvester) an ad hoc statistic Δ*K* based on the rate of change in the log probability of data between successive *K* values (Evanno et al. 2005).

## Results

### Statistical analysis of genomic SSR markers

The number of scorable alleles produced per primer ranged from 1 to 7. Primer SSR02 generated highest number of alleles of 7 and primers UGEP84, UGEP102 and UGEP109 generated least number of allele of 1. The major allele frequency of SSR markers ranged from 0.80 to 0.99 and mean major allele frequency was 0.92. The UGEP84 showed highest major allele frequency of 0.99, while SSR01 showed lowest major allele frequency of 0.80. Totally 252 alleles were produced, of which 186 (73.80 %) were polymorphic with an average of 2.13 alleles per primer and 1.45 alleles per genotype. Out of 87 markers, 72 (82.75 %) were found to be polymorphic. Among polymorphic markers the percentage of polymorphism ranged from 25.0 to 85.71 %; average polymorphism was 59.94 % per marker. Primer SSR10 produced highest polymorphism of 85.71 % and primer UGEP69 produced lowest polymorphism of 25 %. The polymorphic alleles were informative to differentiate the selected genotypes. In Indian genotypes, total number of alleles was 136 with an average of 1.5 alleles per primer and 1.06 alleles per genotype. In non-Indian genotypes, the total number of alleles was 110 with an average of 1.26 alleles per primer and 0.85 allele per genotype. In three unknown genotypes, the total number of alleles was 6 with an average of 2 alleles per genotype. Indian genotypes produced an additional 0.21 allele than non-Indian genotypes.

Gene diversity was in the range of 0.02–0.35 with an average value of 0.14 and gene diversity was found to be highest with the primer SSR01 (0.35), followed by SSR02 and SSR10 (0.33). Forty SSR primers showed more gene diversity than the average value (0.14). The heterozygosity ranged from 0.0 to 0.26 and SSR10 showed highest heterozygosity (0.26), followed by UGEP3 (0.25); average heterozygosity was 0.11, close to 100 % homozygosity. The PIC values ranged from 0.32 to 0.64; the average PIC value was 0.44. Primer SSR01 produced highest PIC value of 0.64 and primers UGEP20, UGEP27, UGEP58, UGEP66, UGEP70, UGEP74 and UGEP84 produced lowest PIC value of 0.32. The inbreeding coefficient value ranged from 0.0 to 1.0 and the average value was 0.34. The highest inbreeding coefficient value was observed with UGEP67, UGEP84 and UGEP87 (1); this confirmed heterozygosity. The SSRs which had heterozygosity value of 0 showed highest inbreeding coefficient value of 1 (Table 2).

**Table 2:** List of 87 genomic SSR primers with polymorphism details, used for the analysis of genetic diversity and population structure of 128 finger millet genotypes collected from various geographical regions of the world

Marker	MAF	AN	GD	He	PIC	IC	Marker	MAF	AN	GD	He	PIC	IC
SSR01	0.80	5.0	0.35	0.23	0.64	0.33	UGEP68	0.90	2.0	0.18	0.15	0.48	0.19
SSR02	0.82	7.0	0.33	0.20	0.62	0.41	UGEP69	0.98	2.0	0.05	0.03	0.35	0.33
SSR06	0.84	3.0	0.29	0.23	0.59	0.23	UGEP70	0.99	3.0	0.02	0.01	0.32	0.67
SSR08	0.85	6.0	0.27	0.19	0.56	0.31	UGEP73	0.97	3.0	0.05	0.04	0.35	0.28
SSR10	0.81	4.0	0.33	0.26	0.62	0.23	UGEP74	0.99	2.0	0.02	0.01	0.32	0.67
UGEP1	0.84	3.0	0.30	0.22	0.59	0.26	UGEP75	0.95	4.0	0.11	0.05	0.40	0.56
UGEP3	0.82	3.0	0.32	0.25	0.61	0.22	UGEP76	0.87	2.0	0.24	0.16	0.54	0.33
UGEP5	0.83	2.0	0.31	0.23	0.60	0.24	UGEP77	0.88	2.0	0.22	0.13	0.52	0.41
UGEP6	0.84	3.0	0.28	0.23	0.57	0.20	UGEP78	0.86	3.0	0.26	0.19	0.55	0.27
UGEP7	0.88	3.0	0.22	0.19	0.51	0.14	UGEP79	0.98	3.0	0.03	0.02	0.33	0.50
UGEP8	0.86	3.0	0.25	0.16	0.54	0.34	UGEP80	0.98	4.0	0.04	0.02	0.34	0.40
UGEP9	0.93	4.0	0.14	0.12	0.44	0.17	UGEP81	0.91	2.0	0.18	0.17	0.47	0.02
UGEP10	0.84	2.0	0.28	0.20	0.57	0.28	UGEP83	0.96	3.0	0.08	0.04	0.38	0.54
UGEP11	0.88	3.0	0.22	0.17	0.51	0.21	UGEP84	0.99	1.0	0.02	0.00	0.32	1.00
UGEP12	0.86	2.0	0.26	0.22	0.55	0.15	UGEP86	0.98	2.0	0.04	0.02	0.34	0.40
UGEP13	0.93	5.0	0.14	0.12	0.44	0.18	UGEP87	0.98	3.0	0.03	0.00	0.33	1.00
UGEP15	0.88	3.0	0.22	0.16	0.51	0.28	UGEP88	0.98	3.0	0.03	0.02	0.33	0.50
UGEP16	0.90	3.0	0.18	0.10	0.48	0.45	UGEP90	0.88	2.0	0.21	0.20	0.50	0.09
UGEP17	0.93	3.0	0.13	0.08	0.43	0.42	UGEP91	0.96	2.0	0.07	0.02	0.37	0.66
UGEP18	0.86	4.0	0.26	0.21	0.55	0.20	UGEP93	0.97	3.0	0.06	0.05	0.36	0.23
UGEP19	0.90	3.0	0.19	0.14	0.48	0.26	UGEP95	0.95	3.0	0.11	0.09	0.40	0.11
UGEP20	0.99	3.0	0.02	0.01	0.32	0.67	UGEP96	0.95	3.0	0.09	0.05	0.39	0.49
UGEP21	0.89	2.0	0.20	0.15	0.49	0.25	UGEP97	0.96	2.0	0.08	0.05	0.38	0.34
UGEP22	0.97	3.0	0.05	0.04	0.35	0.27	UGEP98	0.98	3.0	0.04	0.02	0.34	0.40
UGEP24	0.86	2.0	0.26	0.24	0.55	0.08	UGEP100	0.96	3.0	0.07	0.04	0.37	0.44
UGEP25	0.98	3.0	0.04	0.02	0.34	0.39	UGEP101	0.97	4.0	0.06	0.05	0.36	0.24
UGEP26	0.89	3.0	0.20	0.17	0.50	0.16	UGEP102	0.89	1.0	0.21	0.16	0.50	0.22
UGEP27	0.99	3.0	0.02	0.01	0.32	0.67	UGEP58	0.99	3.0	0.02	0.01	0.32	0.67
UGEP28	0.96	4.0	0.08	0.07	0.38	0.16	UGEP59	0.98	4.0	0.05	0.03	0.35	0.33
UGEP29	0.98	4.0	0.05	0.03	0.35	0.33	UGEP60	0.85	2.0	0.27	0.24	0.56	0.10
UGEP31	0.88	2.0	0.23	0.17	0.52	0.25	UGEP62	0.98	3.0	0.03	0.02	0.33	0.50
UGEP33	0.97	3.0	0.06	0.05	0.36	0.24	UGEP64	0.98	4.0	0.03	0.02	0.33	0.50
UGEP34	0.97	3.0	0.05	0.02	0.35	0.57	UGEP65	0.86	2.0	0.25	0.20	0.54	0.22
UGEP45	0.98	3.0	0.05	0.02	0.35	0.66	UGEP66	0.99	2.0	0.02	0.01	0.32	0.67
UGEP46	0.97	2.0	0.05	0.04	0.35	0.28	UGEP67	0.98	2.0	0.05	0.00	0.35	1.00
UGEP47	0.94	3.0	0.11	0.09	0.41	0.24	UGEP104	0.90	3.0	0.19	0.17	0.48	0.09
UGEP50	0.96	3.0	0.08	0.05	0.38	0.34	UGEP105	0.97	3.0	0.05	0.04	0.35	0.27
UGEP51	0.97	3.0	0.05	0.02	0.35	0.56	UGEP106	0.89	2.0	0.20	0.20	0.50	0.00
UGEP52	0.86	2.0	0.25	0.23	0.54	0.09	UGEP107	0.90	2.0	0.19	0.14	0.49	0.27
UGEP53	0.88	3.0	0.22	0.20	0.51	0.07	UGEP108	0.87	3.0	0.24	0.17	0.53	0.28
UGEP54	0.94	4.0	0.11	0.07	0.41	0.38	UGEP109	0.92	1.0	0.15	0.09	0.45	0.44
UGEP56	0.86	2.0	0.25	0.23	0.54	0.09	UGEP110	0.87	2.0	0.24	0.23	0.53	0.05
UGEP57	0.96	4.0	0.07	0.05	0.37	0.21	UGEP111	0.93	2.0	0.13	0.11	0.43	0.19
UGEP103	0.95	4.0	0.09	0.03	0.39	0.66	Mean	0.92	2.9	0.14	0.11	0.44	0.34

*MAF* major allele frequency, *AN* allele no, *GD* gene diversity, *He* heterozygosity, *PIC* polymorphic information content, *IC* inbreeding coefficient

### Jaccard’s similarity coefficient

The value of Jaccard’s similarity coefficients ranged from 0.011 to 0.836. In UPGMA cluster analysis, the genotypes were grouped into three major clusters viz, *Eleusine coracana* major cluster (*Ec*MC) 1 to *Ec*MC3. Indian genotypes DPI009-04 and GPU-45 were placed in *Ec*MC1. The value of Jaccard’s similarity coefficients was 0.836 between these 2 genotypes; this was the highest similarity value obtained between genotypes in this study whereas the lowest value of 0.011 was observed between genotypes IE-3392 and IE-3470, IE-6221 and IE-6240, IE-4073 and IE-4121, and IE-6165 and IE-6221. Between genotypes IE-2437 and IE-2457 the value was 0.019. The similarity value was 0.773 between Indian genotypes GPU-28 and GPU-45. These genotypes (GPU 28 and GPU-45) are blast resistant local varieties cultivated in Karnataka state, India. The Indian genotypes GPU-26, GPU-28, GPU-45, KRI007-01, KRI1311 and GPU-67 were placed in *Ec*MC1 along with IE-7079 which originated from Kenya.

**Fig. 1:**
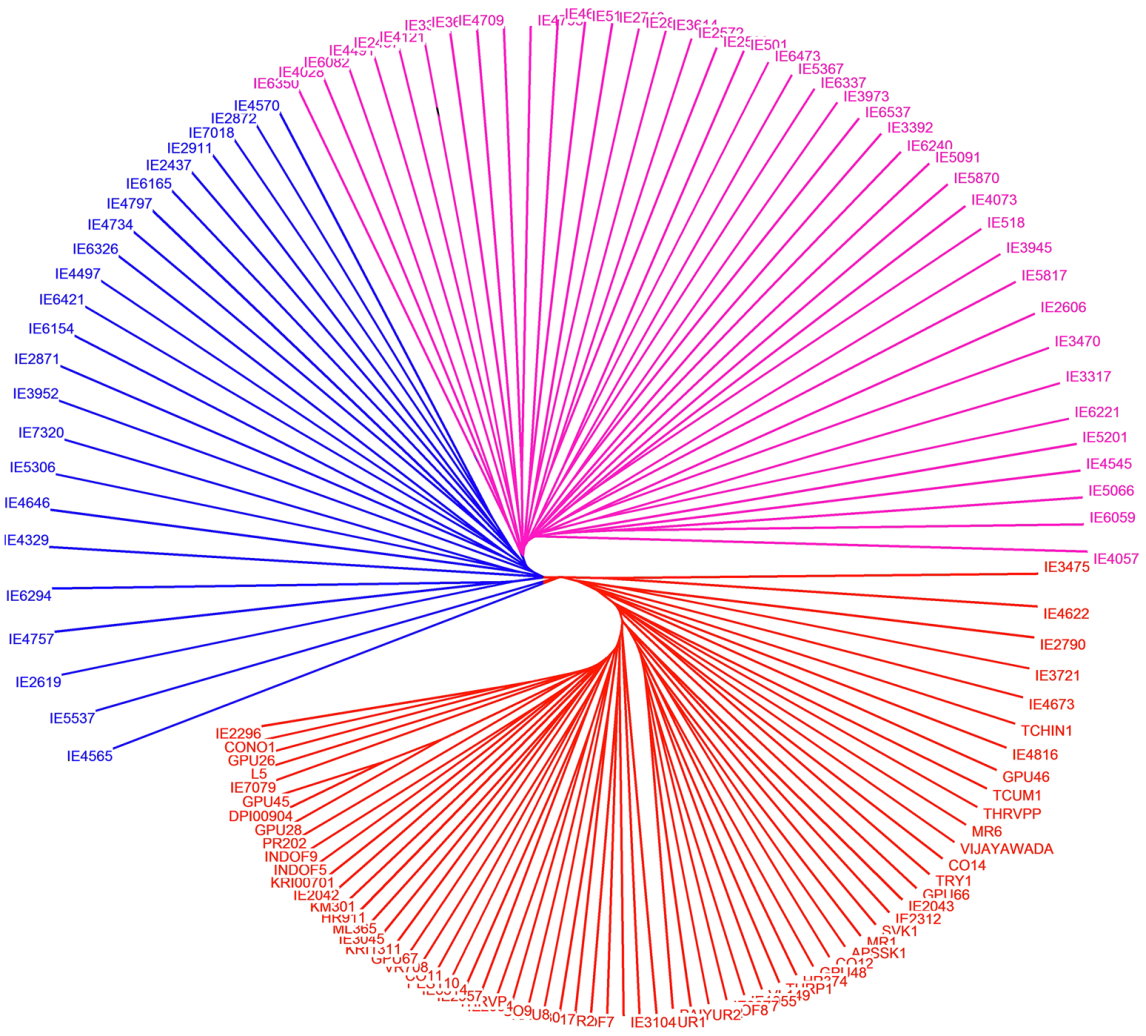
UPGMA cluster analysis generated by Jaccard’s similarity coefficient using 87 genomic SSR markers showing a genetic relationship in finger millet genotypes collected from various geographical regions of the world. *Colors* represent different subpopulations identified in structure analysis as shown in Figs. 3 and 4

Blast susceptible genotypes RAU-8 and CO-9 were placed in *Ec*MC1 and the value of Jaccard’s similarity coefficients between them was 0.449. Another blast susceptible genotype KM-252 was also clustered in *Ec*MC1 with high yielding genotype Paiyur-2; their Jaccard’s similarity coefficients value was 0.547. Out of 64 Indian genotypes, 56 genotypes were placed in *Ec*MC1 along with 6 non-Indian genotypes, IE-2430 and IE-7079 (Kenya), IE-2790 (Malawi), IE-2957 (Germany), IE-3721 (Uganda) and IE-6514 (Zimbabwe) (Fig. 1). This may be due to the fact that the Indian genotypes might have originated from Kenya and Zimbabwe.

The 78.08 % of clusters showed higher bootstrap values with 70–100 % and only 21.91 % of clusters showed lower bootstrap values with 40–69 %. These values confirmed that cluster nodes were well supported and none of the cluster node was found to be poorly supported. Indian genotypes, APSSK-1 and CO-12, THRV-PP and TCUM-1, and CO-14 and TRY-1 showed higher bootstrap values (100 %). Likewise 10 non-Indian genotypes, IE-4570, IE-2872, IE-4795, IE-4709, IE-6059, IE-5066, IE-5817, IE-3945, IE-6240 and IE-3392 showed higher bootstrap values (100 %). Based on the cluster grouping and the bootstrap value (100 %) it has been confirmed that all 64 Indian genotypes are genetically distinct from 61 non-Indian genotypes.

### Statistical fitness analysis

**Fig. 2:**
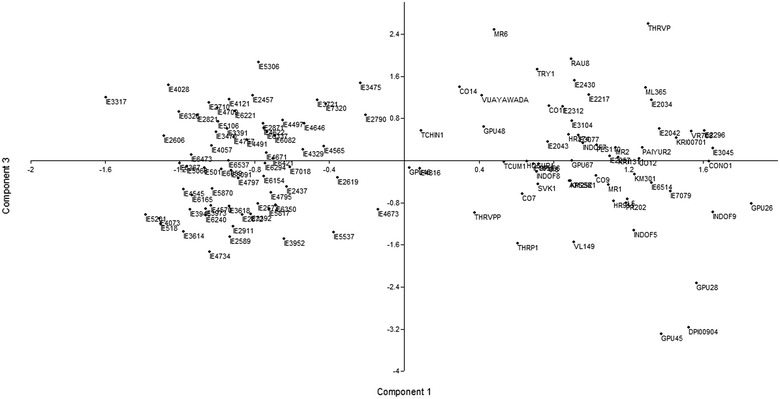
PCA scatter diagram analysis showing the distributions of finger millet genotypes. *Component 1* and *3* are the principal components of first and the third respectively

PCA analysis showed that first and third component axes accounted for 12.08 and 3.21 % respectively of the total variance and eigenvalues were 5.04 and 1.3 respectively. PCA plot was made using the first and third components based on the Var-covar matrix which showed that Indian genotypes GPU-28, GPU-45 and DPI009-04 were closely dispersed in first component axis (Fig. 2). The non-Indian genotype IE-2790 from Malawi has dispersed distantly in third coordinate with genotypes IE-3475 and IE-4673 of Indian origin (close to first coordinate). Other 2 Indian genotypes GPU-46 and IE-4816 were dispersed in first coordinate; however the position of GPU-46 and IE-4816 were close to third coordinate. This result corresponded to dendogram and Jaccard’s similarity coefficients analyses as GPU-28, GPU-45 and DPI009-04 were placed in the same *Ec*MC1. PCA scatter diagram showed that the jolliffe cut off value was 0.16809 and the first 65 principal components (PCs) with eigenvalues greater (5.04957–0.172131) than this cut-off value. PCs associated with the covariance matrix had eigenvalues greater in size than the average of all the eigenvalues showing that PCs were significant. The CCC value was 0.9216 which indicated that the cluster result was very good and acceptable to the genetic similarity matrix calculation.

### Population structure

**Fig. 3:**
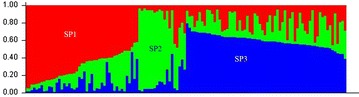
The population structure analysis; the 128 finger millet genotypes were grouped into three subpopulations based on structure analysis

**Fig. 4:**
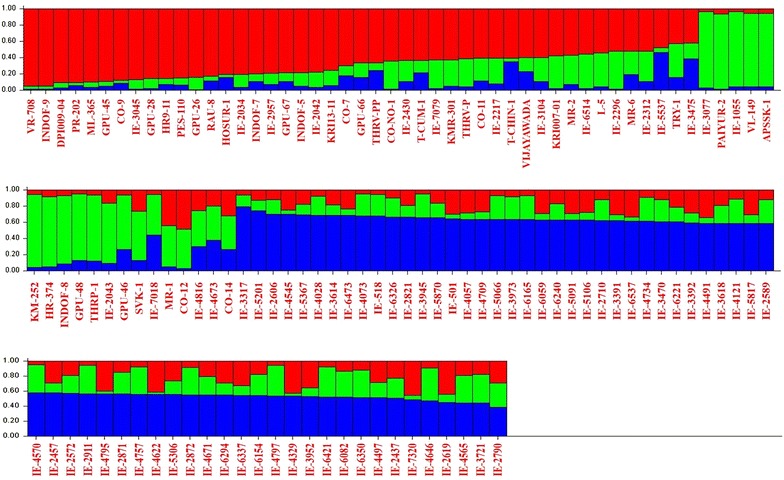
The subpopulations showing admixture of alleles in 128 genotypes of finger millet based on structure analysis

Structure analysis showed that the maximum Δ*K* value observed was *K* = 3 which suggested that the 128 finger millet genotypes were broadly divided into three SPs (SP1, SP2 and SP3) (Fig. 3). The genetic relationship among the SPs provided various confirmations for gene flow between SPs. This group also confirmed the Jaccard’s similarity coefficient analysis which resulted in grouping of finger millet genotypes into three major clusters (*Ec*MC1–*Ec*MC3). Indian genotypes were placed in first two SPs (SP1 and SP2) and non-Indian genotypes were placed in last two SPs (SP2 and SP3). This result confirmed that SP2 had both Indian and non-Indian genotypes and the results of the structure showed that all the three SPs had an admixture of alleles and no pure line was observed. Indian genotypes, VR-708, INDOF-9, DPI009-04, IE-3077 and Paiyur-2 were 85–95 % pure lines (Fig. 4). These genotypes were grouped in SP1 and SP2 and clustered at *Ec*MC1 in UPGMA-Jaccard’s similarity coefficient analysis.

### AMOVA analysis

**Fig. 5:**
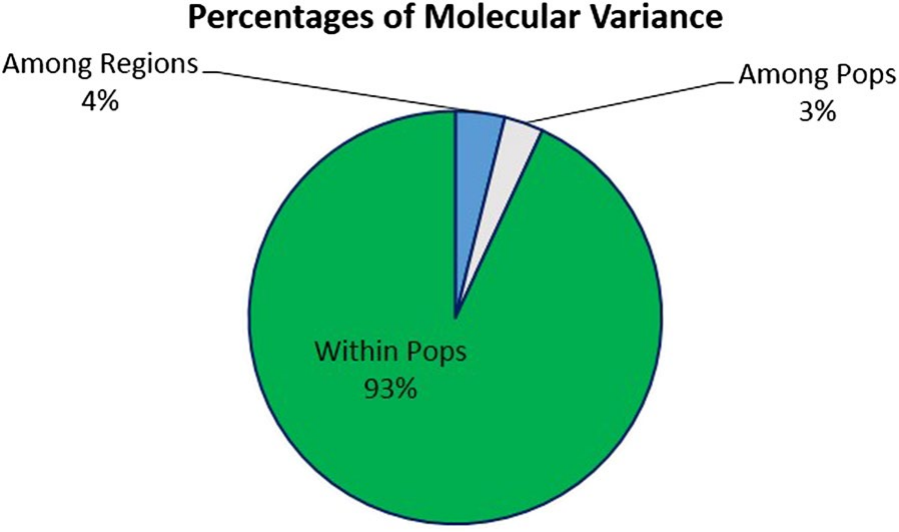
AMOVA analysis showing the percentage of molecular variance among and within populations and among the various geographical regions in finger millet genotypes collected from different geographical regions

Hierarchical analysis of Indian and non-Indian finger millet genotypes’ diversity was performed using AMOVA to know the variation in genotypes from various geographical regions, among populations and within populations. AMOVA analysis showed the degree of freedom (df) value among the various geographical regions, among populations and within populations as 9, 3 and 115 respectively. Sums of squared deviations’ (SS) value among the various geographical regions, among populations and within populations were 588.72, 178.54 and 4539.23 respectively. The means of squared deviation (MS) values among the various geographical regions, among populations and within populations were 65.41, 59.51 and 39.47 respectively. Percentage of molecular variance among the various geographical regions was 4 %; among populations it was 3 % and within populations it was 93 % (Fig. 5). The Phi RT, Phi PR, Phi PT, Phi RT max, Phi ‘RT, Phi PR max and Phi’ PR values were 0.039, 0.032, 0.069, 0.534, 0.072, 0.535 and 0.060 respectively. Phi RT and Phi PT *P* value was 0.001 and Phi PR *P*-value was 0.003; these values were less than 1 which confirmed the AMOVA results. The genotypic diversity value (P) was highly significant (*p* < 0.001) at all the three hierarchical levels (among the various geographical regions, among populations and within populations). The highest value of genetic variation was observed among Indian populations (2722.43) and it was lower (31.0) in the Burundi and Nigerian populations. Among non-Indian genotypes, highest value of genetic variation (712.38) was found in Zimbabwean genotypes followed by Uganda genotypes (308.50). There was good correspondence between the Jaccard’s similarity coefficient, PCA, population structure and the AMOVA in differentiating the finger millet genotypes into different clusters based on their geographical regions.

## Discussion

### Finger millet genotypes

The presented genetic relationships analyses are the first one based on 45 Southern Indian genotypes using 87 genomic SSR markers. PIC value, allele frequency, gene diversity and other indices of all polymorphic markers clearly demonstrated that SSRs could be successfully used for such studies. Our results are in agreement with results presented by others in finger millet as follows: 67 genotypes using 14 genomic SSR markers (Arya et al. 2013); 52 genotypes using 24 genomic SSR markers (Kumar et al. 2012), 79 genotypes using 45 genomic SSR markers (Dida et al. 2008), 83 genotypes using 10 genomic SSR markers (Panwar et al. 2010b).

### Genetic diversity analysis

In general, use of SSR markers in millets to study the genetic diversity is a most appropriate technique providing useful molecular data when compared to the other marker systems and it has increased acceptance world-wide (Stich et al. 2010). In the present study SSR markers showed 73.80 % polymorphism using 87 genomic SSR markers in 128 genotypes. However, out of 87 markers, only 72 markers (82.75 %) were found to be polymorphic. It may be due to the fact that finger millet is a highly self-pollinating crop which might have caused low level of polymorphism in this study. This is in agreement with previous reports of Babu et al. (2014a, b) who reported that 35 (48 %) out of 74 genic SSR primers and 21 (46 %) out of 46 genomic SSR primers were found to be polymorphic in a study using 190 genotypes of finger millet.

In finger millet genotypes only a few reports are available on genetic diversity analysis using SSR markers with good amount of polymorphism. These are; 70.19 % polymorphism in 83 genotypes using 10 genomic SSR markers (Panwar et al. 2010b); 66.6 % polymorphism in 52 genotypes using 24 genomic SSR markers (Kumar et al. 2012), 68.23 % polymorphism in 103 genotypes using 30 genic SSR markers (Nirgude et al. 2014). Similarly, Babu et al. (2014a, b) reported 72.22 % polymorphism using 46 genomic SSR makers and 70.67 % polymorphism using 74 genic SSR markers in 190 genotypes. In the present study we have detected highest polymorphism percentage ranging from 25.0 to 85.71 % with an average of 59.94 % polymorphism; this covered all the chromosomes in all genotypes. The highest polymorphism of 85.71 % was detected with primer SSR10 and lowest polymorphism of 25.0 % was detected with primer UGEP69.

In the present study, genomic SSR markers produced highest average of 1.06 alleles per Indian genotypes of finger millet. Similarly, Panwar et al. (2010b) and Arya et al. (2013) reported an average of 0.84 and 1.02 alleles respectively per finger millet genotype using SSR markers. Also, Babu et al. (2014b) reported an average of 0.7 allele per finger millet genotype using 74 genic SSR markers. The PIC values ranged from 0.32 to 0. 64; average PIC value was 0.44 which showed the ability of genomic SSR markers to discriminate the Indian and non-Indian genotypes. This is in agreement with previous reports using SSR markers in finger millet (Babu et al. 2014b).

The gene diversity values were in the range of 0.02–0.35 with an average value of 0.14. This is in agreement with previous report by Babu et al. (2014a) based on genomic SSR markers in finger millet genotypes. However, based on genic SSRs, Nirgude et al. (2014) observed lower gene diversity values (0.02–0.32) compared to our results. This low amount of gene diversity may be due to the lower number (15) of genic SSR markers they used. Babu et al. (2014b) observed higher amount of heterozygosity ranging from 0.00 to 1.00 with an average of 0.278 in 190 finger millet genotypes using 74 genic SSR markers. This may be due to the fact that lower number of alleles was produced by genic SSR markers. In the present study, we have observed lower heterozygosity value ranging from 0 to 0.26 with an average heterozygosity value of 0.11, which was close to 100 % homozygosity. The inbreeding coefficient value ranged from 0 to 1 and the average value was 0.34. The SSRs which had heterozygosity value of 0, showed highest inbreeding coefficient value of 1. The gene diversity and heterozygosity present in Indian and non-Indian genotypes of finger millet showed that the genomic SSR markers used in the present study were more polymorphic. Similar results were obtained in 155 foxtail millet genotypes by Vetriventhan et al. (2013), using 72 genomic SSR markers where an average heterozygosity of 0.04 was recorded, which was close to 100 % homozygosity.

The average similarity coefficient value was higher in Indian genotypes (0.346) than those in non-Indian genotypes (0.220). Also, similarity coefficient value was higher for those genotypes collected from Southern India with least similarity coefficient value for those from Zimbabwe, Nepal, Uganda and Kenya. This confirmed that the Indian genotypes might have originated from the same breeding population and non-Indian genotypes might have originated from different breeding population. Similarly, Arya et al. (2013) reported higher similarity coefficient value among finger millet genotypes collected from India and the least similarity coefficient value from genotypes of Africa using genomic SSR markers. Also, Bashir et al. (2015) detected higher similarity coefficient in pearl millet using SSR markers.

The bootstrap value was 100 % in the final cluster node which confirmed that all genotypes were genetically diverse. This is in agreement with previous reports by Panwar et al. (2010b) in 83 finger millet genotypes collected from various regions of India and Africa. Similarly, Dida et al. (2008) analyzed 79 finger millet genotypes collected from Africa, Asia, Uganda and Kenya using genomic SSR markers which showed the bootstrap value of 100 % in the final cluster node. Panwar et al. (2010b) reported that CCC value was 0.675 in 83 finger millet genotypes using SSR primers. However, in the present study, we have obtained highest CCC value of 0.9216 which is associated with acceptable genetic similarity matrix. Similarly, Ghasemi Ghehsareh et al. (2015) also obtained highest CCC value of 0.9968 in 53 genotypes representing eight species collected from Iran using microsatellite markers.

The affinities produced by PCA are generally in agreement with the results of the UPGMA cluster analysis. The first and third components axes accounted for 15.29 % of the total variance. The genotypes were distributed according to their geographical regions and especially Indian genotypes were dispersed according to the local site of collection; this suggested that different sites in the PCA plot were good to estimate the genetic diversity. The affinities produced by PCA are generally in agreement with the results of the  structure analysis. Similarly, Koehmstedt et al. (2010) observed that first two components of the PCA produced 24.8 % of total variation using 15 SSR markers among a subset of 99 olive genotypes collected from the United States Department of Agriculture in Davis. Dossett et al. (2012) found that first three eigenvalues produced 9.6 % of variance in 148 genotypes of blackcap using 21 SSR markers. In the present study, we also observed first three eigenvalues of 7.94 %.

The AMOVA analysis showed that the percentages of molecular variance among the various geographical regions, among populations and within populations were 4, 3 and 93 % respectively. Similarly, Babu et al. (2014b) reported molecular variance only within populations (73 %) and among populations (27 %) in 190 finger millet genotypes using 74 genic SSR markers. The present study is the first and detailed report on genetic diversity analysis of 128 finger millet genotypes based on their geographical regions using genomic SSR markers. AMOVA analysis showed significant differences between the genotypes and also produced greater percentage of molecular variance among the geographical regions, among populations and within populations. This was due to the self-pollinating nature of finger millet. It was also proved by population structure analysis that these populations were genetically isolated from each other. There was good correspondence between the AMOVA and the population structure in differentiating the finger millet genotypes into different clusters.

Structure analysis showed maximum D*K* value of *K* = 3; 128 genotypes were divided into three subpopulations (SP1, SP2 and SP3). In the present study using SSR markers all subpopulations had an admixture of alleles and no pure line was observed. This is in agreement with previous report of Dida et al. (2008) who observed an admixture of alleles with African and Asian alleles of finger millet genotypes using 45 SSR markers. Similarly Babu et al. (2014b) also identified four subpopulations among 190 finger millet genotypes using 74 genic SSR makers with an admixture of alleles from other populations and no pure line was observed. SSR marker system has been found to be superior over other markers like RFLP, RAPD, ISSR, and AFLP. In the present study, we have observed that Paiyur-2 had only 95 % purity this may be due to locus specific alleles produced by SSR markers; this was not obtained in previous study using RAPD markers (Ramakrishnan et al. 2015). This study helped to predict the important genotypes with putative agronomic traits. We found that Paiyur-2 is a high yielding genotype cultivated in Southern India and this genotype was found to be 95 % pure line. Population structure corresponded to PCA, AMOVA and Jaccard’s similarity coefficient. Similarly Khadari et al. (2003), Hazarika et al. (2014) and Khan et al. (2014) chose only SSR markers as the markers of choice for breeding research, because of their locus specificity and variability, ease to use, accessibility of detection, reproducibility and data exchange.

In conclusion, we have confirmed that all 128 genotypes were genetically diverse and were clustered into three subpopulations based on their geographic region of origin. Data generated through this study may be utilized for mapping of any important agronomical trait for breeding programes to improve the finger millet.

## Abbreviations

NBPGR: National bureau of plant genetic resources
ICRISAT: International crops research institute for the semi-arid-tropics
AICMMP: All India coordinated minor millet project
SSR: Simple sequence repeats
EST-SSR: Expressed sequence tag- simple sequence repeats
PIC: Polymorphic information content
UPGMA: Unweighted pair-group method with arithmetic average
AMOVA: Analysis of the molecular variation
CCC: Cophenetic correlation coefficient
PCA: Principal component analysis
SPs: Subpopulations
MCMC: Markov Chain Monte Carlo
